# Pleomorphic Dermal Sarcoma of the Scalp

**DOI:** 10.7759/cureus.2979

**Published:** 2018-07-13

**Authors:** Nikolai Klebanov, Mai P Hoang, Bobby Y Reddy

**Affiliations:** 1 Dermatology, Massachusetts General Hospital, Boston, USA; 2 Pathology, Massachusetts General Hospital/Harvard Medical School, Boston, USA

**Keywords:** undifferentiated pleomorphic sarcoma, pleomorphic dermal sarcoma, atypical fibroxanthoma

## Abstract

Pleomorphic dermal sarcoma (PDS) is a rare mesenchymal tissue tumor. Distinguishing PDS from similar conditions, such as atypical fibroxanthoma (AFX), its less aggressive tumor counterpart, is difficult, as they are clinically and histologically similar. We present a case of a 77-year-old man presenting with a large nodular scalp lesion of three weeks duration. Pathology revealed a 3.3 cm invasive pleomorphic dermal sarcoma. Surgical excision with 2 cm margins was performed with successful healing of the graft. This case highlights a rare case of a large pleomorphic dermal sarcoma and discusses the histological features and management of PDS.

## Introduction

Atypical fibroxanthoma (AFX) and pleomorphic dermal sarcoma (PDS), formerly known as malignant fibrous histiocytoma (MFH), are rare mesenchymal tissue tumors. These tumors are clinically and morphologically similar, and it is debated whether PDS represents a separate clinical entity from AFX or it is merely a more aggressive variant. AFX occurs more frequently than PDS; however, both of these entities are quite uncommon. AFX cases have been moderately well-documented, thus allowing for a study of the risk factors and the genetics of these mesenchymal malignancies.

AFX tumors affect elderly patients (usually male patients between the ages of 70 and 80) [1-2], favor ultraviolet (UV)-damaged skin of the head and neck, and are associated with several known risk factors, including radiation (including UV), xeroderma pigmentosum, and a history of organ transplantation [3-8]. On histological examination, AFX lesions are characterized by pleomorphic tumor cells, atypical spindle cells, and giant cells. The genetic underpinnings of AFX are poorly understood given the rarity of these tumors. Case series have identified UV-signature mutations in the TP53 gene [9-10] as well as likely oncogenic HRAS, KRAS, NOTCH1/2, and FAT1 mutations [7,11] by the sequencing of AFX tumor samples. Given the predilection of AFX for the sun-exposed skin of the head and neck in older patients and the presence of UV-signature p53 mutations, AFX emerges as a tumor with similar characteristics to non-melanoma skin cancers, such as basal cell carcinoma (BCC) and squamous cell carcinoma (SCC) (which are currently thought to be attributable to cumulative and chronic sun exposure). In fact, a personal history of BCC or SCC is frequently identified in patients presenting with AFX [12-13].

Distinguishing AFX from PDS is vital, given that PDS is a much more clinically aggressive tumor, requiring prompt diagnosis and decisive treatment. However, clinically distinguishing the two cutaneous diseases is often difficult because of the similarities in their gross and microscopic morphologies. Several studies support the treatment of AFX with Mohs surgery [12-15] but less is known about the treatment of the more aggressive PDS variant.

We present a case of a mesenchymal tissue tumor affecting the scalp of an elderly male patient. We discuss the pathological diagnosis of the patient’s tumor and the successful surgical management of the lesion. Our patient’s case illustrates the rare pleomorphic dermal sarcoma malignancy. This case may help clinicians be more aware of the related atypical fibroxanthoma (AFX) and pleomorphic dermal sarcoma (PDS) mesenchymal tumors and of the surgical management that can be used for the management of these rare conditions.

## Case presentation

**Figure 1:**
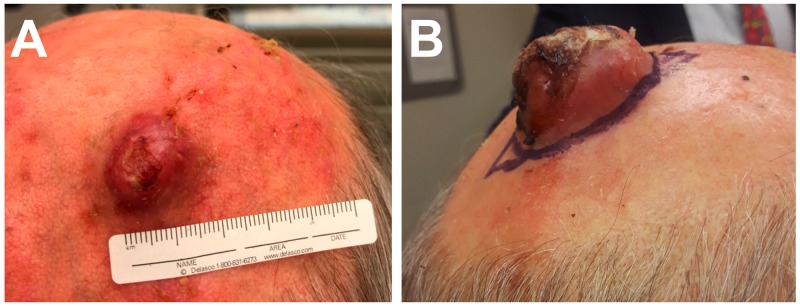
Gross scalp lesion morphology 1A. Cutaneous 1.5 cm nodule at initial visit three weeks after the patient initially noted the lesion. 1B. Erythematous ulcerated nodule prior to the wide, local excision on the third week following initial clinic presentation.

A 77-year-old man with no history of skin cancer presented to the outpatient dermatology clinic for a scalp lesion of three weeks duration. He reported rapid lesion growth, but no change in the overall appearance nor any associated symptoms. Examination revealed a mobile, round, exophytic nodule with overlying ulceration and hemorrhagic crust, approximately 1.5 cm in diameter (Figure 1A). Given a high clinical suspicion of malignancy, the lesion was excised three weeks following the initial visit using a fusiform (elliptical) incision with 1 cm margins. The lesion immediately prior to the procedure is seen in Figure 1B.

**Figure 2:**
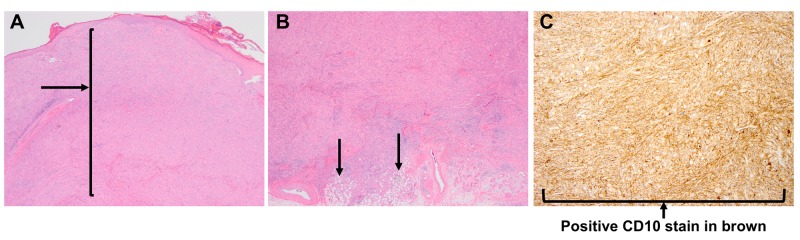
Histological tumor features 2A. An expansile tumor in the dermis (original magnification X40). 2B: Involvement of subcutaneous fat is noted (original magnification X40). 2C: Diffuse CD10 expression by the tumor cells (original magnification X100).

A histopathologic examination of an excisional biopsy of the cutaneous lesion revealed a proliferation of spindle and pleomorphic tumor cells, which flattened the overlying epidermis (Figure 2A) and extended to the subcutaneous tissue (Figure 2B). The tumor cells were large and polygonal and contained eosinophilic cytoplasm. They expressed diffuse CD10 positivity (Figure 2C) and focal CD68 positivity. The tumor cells were negative for p40 and SOX10, excluding the possibility of sarcomatoid carcinoma and melanoma, respectively. The histopathologic findings were found to be consistent with invasive pleomorphic dermal sarcoma. The tumor measured 3.3 cm at the largest dimension, and tumor cells were found to be present at the surgical margin. There was no evidence of lymphovascular or perineural invasion.

**Figure 3:**
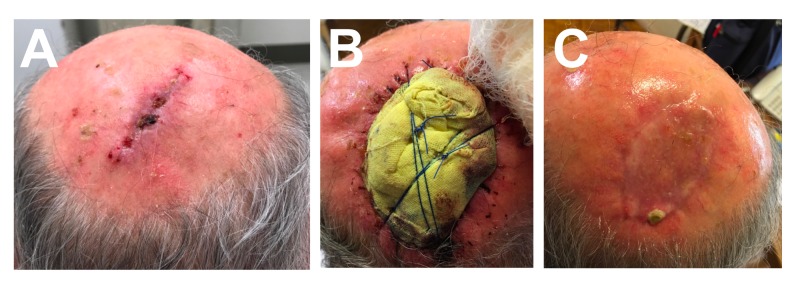
Outcomes of surgical lesion management 3A. Surgical wound two weeks following initial narrow elliptical excision. 3B. Petrolatum gauze scalp dressing applied following the excision with wide (2 cm) surgical margins. 3C. Healthy full-thickness skin graft healing two weeks following the wide, local excision.

A surgical, wide, local excision was planned. Preparatory computed tomography (CT) imaging revealed an approximately 3.9 cm focus of enhancement with central ulceration in the soft tissue of the posterior scalp and confirmed a lack of bony erosion in the underlying calvarium. No metastatic adenopathy was appreciated. Figure 3A demonstrates the scalp vertex prior to surgery. A wide local excision was performed using a 2 cm margin around the remaining tumor for a 7.5 cm area of planned excision. The repair utilized a 7x3.5 cm full-thickness skin graft from the left upper chest. The immediate post-surgical course was uncomplicated. Wound care with sterile petrolatum gauze dressing (Figure 3B) sutured to the scalp for two weeks helped with successful graft healing (Figure 3C). At the one-month follow-up after the operation, a review of systems did not reveal systemic relapse and an examination of the surgical scar and cervical lymph nodes did not reveal local recurrence. The patient will continue to have close monitoring by the dermatology team twice per year.

## Discussion

Given the high risk of malignant behavior in pleomorphic dermal sarcoma (PDS) tumors, it is important to distinguish PDS from its more benign atypical fibroxanthoma (AFX) counterpart. This decision is made difficult by the morphological similarities between AFX and PDS, such as spindle or round cell histology, the presence of pleomorphism and atypical mitotic figures, and CD10 marker positivity. PDS sometimes reveals more aggressive histological features than AFX, such as evidence of deeper subdermal involvement, the presence of lymphovascular or perineural invasion, and/or necrosis [7].

In the case presented here, the histological examination did not reveal any evidence of lymphovascular or perineural invasion. Likewise, as diffuse CD10 positivity is observed in both AFX and PDS tumors, CD10 positivity alone could not be used to make the diagnosis of PDS. However, the involvement of the deep subcutis favors a diagnosis of PDS [3] and given the gross size of the tumor and the depth of penetration involving the subcutaneous fat (Figures 2A-2B), there was a low threshold to treat this growth aggressively as a pleomorphic dermal sarcoma. In the literature, reported treatments for atypical fibroxanthoma (AFX) include cryotherapy, radiation, and surgical methods: wide local excision and Mohs micrographic surgery. Surgical excision is preferred over cryotherapy and irradiation. Cryotherapy is not recommended due to the risk of recurrence or metastasis, and irradiation poses the risk of causing tumor DNA dysregulation, resulting in progression to a more aggressive tumor [16]. A meta-analysis of 23 studies comparing treatment with Mohs micrographic surgery to excision for atypical fibroxanthoma suggested that Mohs surgery was associated with a lower recurrence rate than wide local excision [15].

Less is known overall regarding PDS management given the tumor’s rarity. Complete surgical excision is considered as the first line option for PDS management [17], and an incomplete excision places the patient at increased risk of local recurrence [18-19], with the study by Tardío et al. reporting a 20% rate of local recurrence after surgical management, all in patients with incomplete resections. Although the metastatic potential of this rare tumor is estimated to be lower than 5% [19-20], given the poor prognosis of untreated PDS, it is vital for clinicians to be aware of this tumor and proceed to prompt surgical management, ensuring clear margins.

## Conclusions

Pleomorphic dermal sarcoma is a rare tumor affecting mesenchymal tissue. We present the case of pleomorphic dermal sarcoma occurring on the scalp of a 77-year-old male patient. Successful treatment with a wide, local excision was performed. This case highlights the morphologic and histologic characteristics of PDS and discusses what is known about the common treatments of rare mesenchymal malignancies such as atypical fibroxanthoma (AFX) and PDS.
